# Diversity of endoscopy center operations and practice variation across California’s safety-net hospital system: a statewide survey

**DOI:** 10.1186/1756-0500-6-233

**Published:** 2013-06-15

**Authors:** Lukejohn W Day, Taft Bhuket, John M Inadomi, Hal F Yee

**Affiliations:** 1 Division of Gastroenterology, San Francisco General Hospital and Trauma Center, San Francisco, CA, USA; 2 Department of Medicine, University of California, San Francisco, CA, USA; 3 Department of Medicine, Division of Gastroenterology, Alameda County Medical Center, Oakland, CA, USA; 4 Department of Medicine, Division of Gastroenterology and Hepatology, University of Washington, Seattle, WA, USA; 5 Los Angeles County, Department of Health Services, Los Angeles, CA, USA; 6 San Francisco General Hospital, 1001 Potrero Avenue, 3D-5, San Francisco, CA 94110, USA

**Keywords:** Endoscopy, Health care disparities, Practice variation, Safety net hospital, Access to medical care

## Abstract

**Background:**

Little is known about endoscopic services provided or operational practice variation within California public hospital endoscopy centers.

**Methods:**

A survey was distributed to all 18 California public hospitals with endoscopy centers to assess operational practices.

**Results:**

Eight of 18 hospitals responded to the survey. Six of the eight responding hospitals used a closed access system for patient referrals. Mean wait time for an endoscopic procedure was 42.4 ± 37.7 days (N = 8) with a mean procedure no-show/cancellation rate of 14.5 ± 8.0% (N = 7). All responding public hospitals performed colonoscopy, esophagogastroduodenoscopy, PEG tube placement, and endoscopic retrograde cholangiopancreatography (ERCP) with two hospitals performing endoscopic ultrasound. There was significant practice variation in the documentation of endoscopic quality and performance measurements among the responding hospitals. Multiple methods were used to communicate pathology results to patients: GI clinic visit (6/8), primary physician (4/8), telephone (2/8) or letter (1/8).

**Conclusion:**

Our study highlights the diversity and practice variations of endoscopy center operations at California public hospitals and serves as a catalyst for future collaborations among safety-net hospitals.

## Background

Gastroenterology (GI) consultations and the number of endoscopic procedures performed have dramatically increased over the last decade [1-3]. This increase has been most notable in safety-net hospitals whereby GI is the most frequently requested specialty service [2]. This rising demand has contributed to problems with timely access to specialized GI care. For example, medical directors of public hospitals in California report a difficult time obtaining specialized care for 80% of uninsured patients as compared to just 5% for patients with private insurance [4]. However, it is unclear what factors may be contributing to such a striking disparity and whether or not practice variation may be playing a prominent role.

Practice variation is prevalent throughout the American healthcare system and contributes to the rising costs of healthcare, inefficiency within the system, and can impede the delivery of high quality care [5]. Practice variation has been reported in GI, with wide variation noted in the performing of colonoscopy [6,7], management of inflammatory bowel disease [8], and treatment of chronic illnesses [9-15]. Yet, scant literature exists on the practice patterns of endoscopy centers with respect to operations and services offered [16] and of the limited data available most have focused on ambulatory endoscopy centers. Such information is invaluable to have in order to help elucidate potential barriers to GI care that may exist and is essential to have for the growing safety-net hospital system.

To date no study has assessed the endoscopic services available, practice variation within or the organizational structure of endoscopy centers in safety-net hospitals. The goal of our study was to assess the current GI endoscopic services provided, examine practice variation with respect to operations and to study barriers that may exist at providing endoscopic care at the 18 public and not-for profit hospitals, academic medical centers and comprehensive health care systems throughout California that provide medical care to over six million underserved patients.

## Methods

### Study design

An electronic based survey was developed to study the organizational structure and practices of endoscopy centers in the California safety-net hospital system and was administered to endoscopy center directors at California public hospitals from May 8, 2010 to August 1, 2010. For the purposes of this study a public hospital is defined as a not-for-profit hospital operated and supported by a city, county, special district or state government.

### Survey development

Few surveys have been developed or validated to assess the endoscopic services provided or organizational design of endoscopy centers [16] and none have focused on public hospitals. Consequently, using previously published surveys as a foundation, a survey was initially developed by L.W.D. to assess the endoscopic services and capacity of GI endoscopy centers at California public hospitals. The initial survey was divided into five sections: endoscopy center demographics, types of endoscopic services provided and volume of services, endoscopy center staffing ratios, specific quality performance metrics and a qualitative assessment of endoscopy center directors’ views on factors that limit increasing endoscopic volume at public hospitals. After this initial survey was developed a group of five GI experts who manage California public hospital endoscopy centers was convened to form an advisory committee to review and pilot test the survey questions. At the advisory group meeting members believed that is was critical to focus the survey on five key aspects of an endoscopy center: pre-endoscopic procedure factors with particular focus on types of scheduling systems (open versus closed access), type and number of endoscopic services provided, staffing ratios with a focus on providers and nurses, quality metrics (adenoma detection rate, cecal intubation rates, quality of bowel preparations, withdrawal time, adverse rates), performance metrics (percentage of procedures that start on time, room turnover time, procedure time, wait time for endoscopy, no-show/cancellation) and post-endoscopic procedure follow-up with respect to communication of pathology results to patients. Based on the advisory group’s recommendations the survey was then subsequently revised. The revised survey was then administered to the advisory group whereby the format and language of questions was reviewed. The advisory group then discussed the survey via conference call and unanimously agreed upon the questions, wording of and instructions for the questions and format of the survey. The survey was then finalized (Additional file 1) and a method for administration of the survey was then agreed upon by the group through unanimous consensus.

### Study setting and participants

The survey was electronically distributed using SurveyMonkey® to endoscopy center directors at each of the eighteen California public hospitals on May 8, 2010. The eighteen public hospitals were identified by their membership in the California Association of Public Hospitals (CAPH) which was accessed at http://www.caph.org. CAPH represents public hospitals, health care systems and academic medical centers in 15 counties where more than 81% of Californians reside. Its members comprise the core group of health care providers that make up the state’s medical safety-net system. CAPH public hospitals were selected as they provide over half of all hospital care to the state’s 6.7 million uninsured and provide 69% of their care to patients who receive Medi-Cal benefits or are uninsured. Participation in the survey was voluntary and no incentive was offered to participants. In order to maximize response rates a follow-up email was directly sent to each endoscopy center director on May 28, 2010. Lastly, among the remaining hospitals that did not respond an email was again sent to the director of endoscopy as well as the chairperson of the Gastroenterology Division on July 1, 2010. The survey was closed on August 1, 2010.

### Statistical analysis

The mean and standard deviation were calculated for selected categories that contained continuous data. Proportions were provided for the remaining categories that utilized nominal data. All calculations were performed on Stata 11.0 (Stata Corp®, College Station, Texas).

### Ethical considerations

Given our study was related to quality improvement, did not include testing the safety and efficacy of a drug or device in a human subject, and no personal health information was collected at any time formal institutional review was not required per the policy of the University of California San Francisco Committee on Human Research.

## Results

Eight survey responses were received from seventeen hospitals offering gastroenterology endoscopy services in the California safety-net hospital system. These services were no longer offered at Rancho Los Amigos National Medical Center.

### Patient access to endoscopic care

Most endoscopy centers (6/8) in California public hospitals required their patients to attend a GI clinic appointment prior to scheduling an endoscopic procedure (i.e. closed access system). While a closed access system for patient referral was frequently used by California public hospitals, wait times and no-show/cancellation rates varied across hospitals. The shortest wait time was 6 days with the longest being 120 days (mean 42.4 ± 37.7 days). The mean procedure no-show/cancellation rate among responding hospitals was 14.5 ± 8.0% with some hospitals having one-third of their patients cancel/no-show for their procedure appointment. A summary of responses from California public hospital endoscopy center directors is illustrated in Table 1.

**Table 1:** Summary of survey responses from endoscopy centers at California public hospitals

**General characteristics**	
Mean wait time for an endoscopic procedure (days) (N = 8)	42.4 ± 37.7
No show/cancellation rate for endoscopic procedure (%) (N = 7)	14.5 ± 8.0
**Pre-procedure**	
Patient referral (N = 8)	
Open access endoscopy	2 (25.0)
Closed access endoscopy	6 (75.0)
**Procedure**	
Providers performing endoscopy (N = 8)^a^	
Physician assistant	0
Nurse practitioner	2 (25.0)
Family practice/Medicine/Surgery resident	3 (37.5)
Surgeon	4 (50.0)
Gastroenterology fellow	6 (75.0)
Administration of sedation (N = 8)	
Nurse anesthetist	0
Anesthesiologist	1 (12.5)
Nurse	2 (25.0)
Gastroenterologist	5 (62.5)
**Post-endoscopy**	
Delivering pathology results (N = 8)^a^	
Letter	1 (12.5)
Telephone call	2 (25.0)
Follow-up with referring physician	4 (50.0)
Return appointment to GI clinic	6 (75.0)
**Quality measurements (N = 6)**^**a**^	
Adenoma detection	0
Withdrawal time	1 (16.7)
Quality of bowel preparation	2 (33.3)
Cecal intubation	3 (50.0)
Adverse events	6 (100.0)
**Performance measurements (N = 7)**^**a**^	
% of start-on time procedures	1 (14.3)
Room turnover time	1 (14.3)
Procedure duration	2 (28.6)
Wait time for endoscopic procedure	2 (28.6)
No show/cancellation	4 (57.1)
Procedure volume	7 (100.0)

^a^ More than one method/measurement could have been selected.

### Capacity to perform and offer endoscopic procedures

A variety of providers perform endoscopic procedures at California public hospitals. All hospitals had gastroenterologists perform endoscopic procedures but additional medical providers were also involved. Four of the eight hospitals had general surgeons perform endoscopy. Trainee involvement in endoscopic procedures was present in most of the safety-net hospitals; three quarters of respondents (6/8) had GI fellows assist in endoscopic procedures and one-third had resident involvement (family practice/medicine/surgery) (3/8). Lastly, one-quarter of California public hospitals employed non-physicians (i.e. nurse practitioners) to perform endoscopy. In addition to performing endoscopy, gastroenterologists were primarily responsible for administering sedation to patients for a procedure. General anesthesia was infrequently required for endoscopic procedures (6.9%) with the majority of these cases being for advanced endoscopic procedures (endoscopic retrograde cholangiopancreatography (ERCP) and/or endoscopic ultrasound (EUS)).

**Figure 1:**
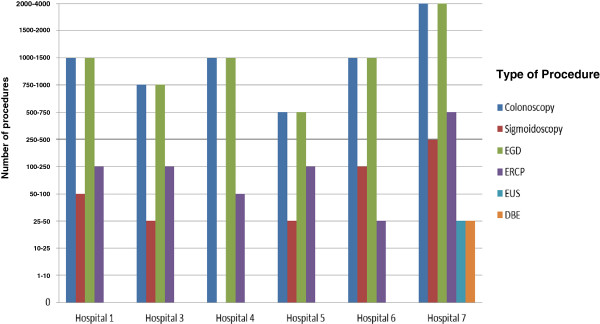
**Volume distribution of various endoscopic procedures performed at California public hospitals.** Note: One hospital did not capture data on the volume of procedures performed.

**Figure 2:**
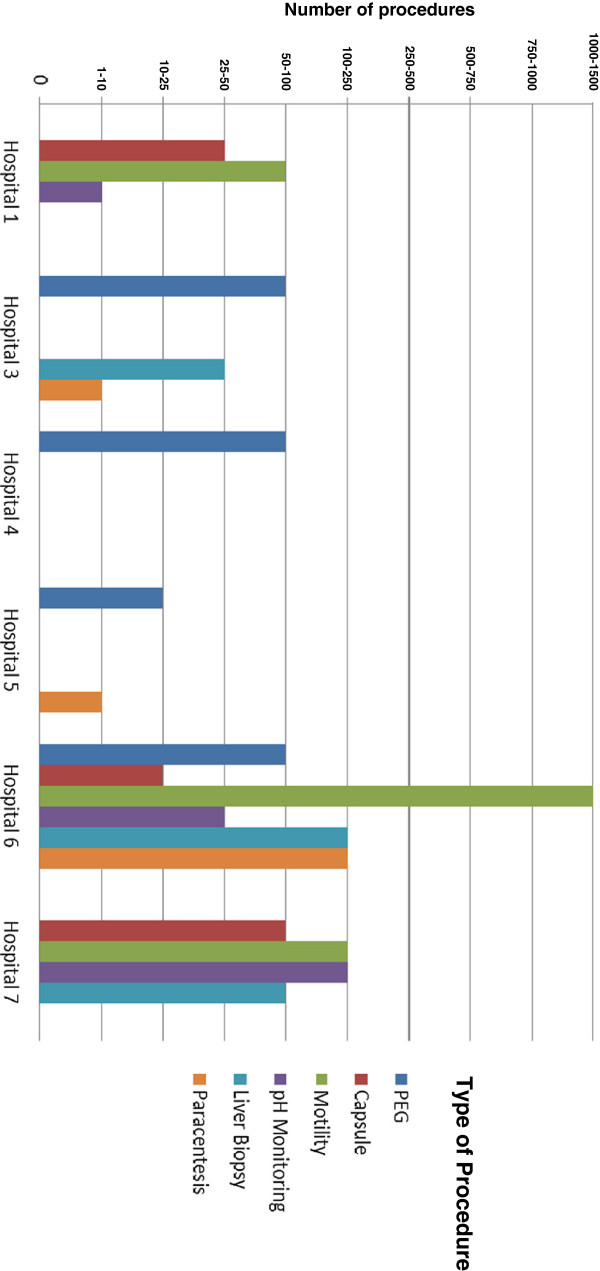
**Volume distribution of other gastrointestinal procedures performed at California public hospitals.** Note: One hospital did not capture data on the volume of procedures performed.

California public hospitals offered and performed an array of GI endoscopic procedures. All responding public hospitals performed colonoscopy, flexible sigmoidoscopy, esophagogastroduodenoscopy (EGD), ERCP and percutaneous endoscopic gastrostomy (PEG) tube placements. Half of the responding hospitals (4/8) performed therapeutic paracentesis, percutaneous liver biopsy, and video capsule endoscopy. Advanced procedures such as EUS and balloon assisted enteroscopy (single or double) were the least frequently offered procedures; only two hospitals performed these procedures. Figures 1 and 2 illustrate the varying procedure volume at the responding hospitals.

A multitude of staffing models and facility layouts were noted across the California safety-net hospital endoscopy centers. All hospitals employed physicians and registered nurses, but one quarter also employed licensed vocational nurses, nurse practitioners, or GI technicians at the endoscopy center. The mean full time equivalent (FTE) for physicians at responding hospitals was 2.5 physicians and 6.2 for registered nurses. At the same time, facility utilization at the endoscopy centers also differed. The pre-procedure area (i.e. where a patient waits prior to their endoscopic procedure) and recovery room shared a combined space among 62.5% of respondents. The mean number of patients that could occupy a pre-procedure area at a given time (i.e. space that included chairs/gurney/bays that could accommodate patients) in responding endoscopy centers was 6.5 ± 3.0 patients. At the same time the mean number of patients that could occupy a recovery area at a given time (i.e. space that included gurney/bays that could accommodate patients) was 8.0 ± 3.0 patients. In 6/8 of responding public hospitals information with regards to endoscopy center expenditures including supplies, equipment purchases, equipment leases, and personnel was not known to endoscopy center directors.

### Measuring performance and quality of endoscopic procedures

While most of the responding public hospitals tracked information about volume and utilization, there was great variation in the documentation of established endoscopic quality and performance measurements. Of the seven responding endoscopy center directors, all recorded information on endoscopic procedure volume, over one-half had data on no-show/cancellation rates for procedures and one-quarter had information on wait time and procedure duration. With respect to colonoscopy quality indicators, six respondents had documentation on the number and type of endoscopic adverse events with three endoscopy centers collecting information on cecal intubation rates, two centers had data on bowel preparation and one center collected data on withdrawal time. None of the participating public hospitals had data on adenoma detection rates.

### Post endoscopy communication with patients: pathology results

Lastly, multiple methodologies were used to communicate pathology results to patients after an endoscopic procedure. Three public hospitals used more than one method to distribute pathology results to their patients. A majority of public hospitals had patients return to a scheduled GI clinic appointment for their pathology results. The remaining modalities utilized were as follows: patient followed up with their primary physician/referring physician (4/8), telephone contact (2/8) or letter from the endoscopy center (1/8).

## Discussion

To our knowledge this is the first systematic examination of the operational systems of a large contingent of public hospital endoscopy centers. Our survey demonstrated several similarities among hospitals in the California safety-net especially in the areas of patient referrals, using training fellows for performing endoscopy and in the offering of specific types of endoscopic procedures. Conversely, significant heterogeneity and practice variation was observed in the performing of advanced endoscopic procedures, recording of performance and quality measurements, and communicating post-endoscopy pathology results to patients. Through this study several barriers to offering, delivering, and following up endoscopic care were identified and are explored and discussed in more detail below.

### Barriers to access for endoscopic care

One interesting finding among the responding endoscopy centers was that three-quarters utilized a closed access system for patient referrals, which is in contrast to what is commonly practiced in the community. There has been considerable debate in the GI literature on the optimal method for referring patients for a GI procedure [17-23], but little focus has been placed on what type of referral method to use within various GI clinical settings, especially within the safety-net hospital system. Proposed methods center on open versus closed access endoscopy. As evidenced in our survey, underserved patients wait a significant amount of time prior to their endoscopic procedure (mean wait time of 42.4 days). Reducing wait times for endoscopic procedures would improve patient satisfaction and increase access to care for underserved patients. This is perhaps why open access endoscopy is an attractive option in that patients can be directly referred for a procedure without prior GI consultation. Cancellation rates are similar between both methods [17] and satisfaction between both is similar [18,19], yet open access yields more inappropriate indications for procedures with a reported 10-50% prevalence of incorrectly/inappropriately scheduled procedures [20-23]. Both methods have advantages and disadvantages within a public hospital system and until now it was unclear the practices of public hospitals. On one hand, open access endoscopy requires a strong infrastructure in place and additional staff to contact and educate patients which may be costly in a financially strapped healthcare system. Conversely, a closed access endoscopy system has its challenges including additional financial commitment of patients for multiple encounters, lost work time and added time of patients visiting clinic prior to scheduling an endoscopy. No comparison between the two methods has been directly made with respect to GI endoscopy centers that serve underserved patients and is an area that deserves further attention.

### Barriers to measuring quality and performance of endoscopy

Our survey also illustrated that public hospital endoscopy centers do not consistently track or document performance and quality metrics and have varying practices. Currently, there are some mechanisms in place that require this documentation such as the Health Plan Employer Data and Information Set [24] and the Centers for Medicaid and Medicare Services [25]. Current and future requirements have important ramifications for public hospitals as they are also held accountable to the tax payers in the region for which they provide care. In examining both performance and quality measurements there was a striking difference in what centers did and did not record. Reasons for these inconsistencies may be related to inadequate information technology infrastructure, lack of staffing resources, and no accepted and/or established measurements of quality and performance. Our findings stress the need for more uniform guidelines on quality and performance measurements in endoscopy and improved methods of documenting such information.

### Barriers to capacity for offering and performing endoscopy

Uniformly, all responding hospitals offered standard endoscopic procedures such as colonoscopy and EGD as well as advanced procedures such as ERCP and PEG tube placement. Yet, there was considerable variability in other services provided such as EUS and video capsule endoscopy. In recent years, both of these advanced endoscopic services have expanded beyond the tertiary referral center and are increasingly integrated into community GI practices. Their clinical usefulness has been well documented in diagnostic modalities such as evaluating iron deficiency anemia [26,27], Crohn’s disease [28], evaluation of pancreatic head and cystic lesions [29] and choledocholithasis [30-32] and in some cases may be superior to standard endoscopic methods. However, our results illustrate that EUS and capsule endoscopy are not available in some California public hospitals. Both modalities require further expertise and training on the part of the gastroenterologist, specialized equipment, and additional staffing and time; resources that a public hospital may not be able to provide. We were not able to address how these additional services were met by hospitals that did not offer them. Some possible explanations include contracting with outside private community GI physicians or large tertiary referral centers. Recognizing these solutions may provide a unique opportunity whereby local GI divisions of public hospitals may be able to pool financial resources and staffing expertise together and create dedicated centers that offer more specialized GI services for underserved patients.

### Barriers to post-endoscopy patient communication

Lastly, our survey offered insight into how pathology results were communicated to underserved patients. While it is highly advocated that such information be communicated to patients [33] there is scant literature on the most effective method by which to convey such information, especially considering the additional constraints present in communication with underserved patients. These constraints may be reflected in that over 75% of survey respondents required their patients to return to GI clinic for their pathology results, with a smaller number using adjunctive methods. Language barriers, low education level, lack of access to telephones or the internet, and lack of housing are all potential barriers to communicating post-endoscopy pathology results to patients in the public hospital setting. Future studies that examine the dissemination of information and in particular endoscopic and pathology results to underserved patients are needed.

There were several limitations to our study. First, we conducted a survey that has not been validated by previous research. Second, our response rate of 44.4% while low is not outside of the response rate for surveys of this type. Studies have illustrated a survey response rate of 37-47% after two to three attempts with no monetary incentive offered [34,35] in a model similar to the one we applied. Third, characteristics of the ten hospitals that did not respond could not be obtained as the survey was anonymous and thus we could not determine if non-responders were systematically different to the responders. Lastly, our study only focused on California public hospitals. Expanding the survey to include other geographic regions of the U.S. would offer a broader picture of other safety net hospital systems and allow us to make comparisons among various state healthcare systems.

## Conclusion

In summary, our survey of California public hospital endoscopy centers offers a unique view of not only the endoscopic services provided in the state but also a closer look at the operational aspects of delivering endoscopic care to underserved patients. While similarities exist between these endoscopy centers in the California safety-net hospital system, there appears to be great variation with respect to procedure volume and advanced procedures, pre-procedure practices and post-procedure follow-up. Our study helps to highlight the diversity of endoscopy center practices and operations of California public hospitals and potentially serves as a catalyst for future collaborations among safety-net hospitals in order to optimize limited resources and services.

## Competing interests

None of the authors have any financial or non-financial competing interests to declare in relation to this manuscript.

## Authors’ contributions

LWD was involved in the conception and design of the study, analysis and interpretation of the data of the article, critical revision of the article for important intellectual content, and provided final approval of the article. TB was involved in the analysis and interpretation of the data of the article, critical revision of the article for important intellectual content, and provided final approval of the article. JI provided critical revision of the article for important intellectual content and final approval of the article. HFY was involved in the conception and design of the study, analysis and interpretation of the data of the article, critical revision of the article for important intellectual content, and provided final approval of the article. All authors read and approved the final manuscript.

## Authors’ information

Lukejohn Day (conception and design; analysis and interpretation of data of the article; writing of the manuscript, critical revision of the article for important intellectual content; final approval of the article).

Taft Bhuket (critical revision of the article for important intellectual content; final approval of the article).

John Inadomi (critical revision of the article for important intellectual content; final approval of the article).

Hal Yee (conception and design; analysis and interpretation of data of the article; critical revision of the article for important intellectual content; final approval of the article).

## Supplementary Material

Additional file 1 Survey distributed to California public hospitals (See attached document). Click here for file
