# Supplementary material for: Diversity of endoscopy center operations and practice variation across California’s safety-net hospital system: a statewide survey
Source: BMC Res Notes. 2013 Jun 15;6:233. doi: 10.1186/1756-0500-6-233 (PMC3693938; doi:10.1186/1756-0500-6-233)
Supplement: Additional file 1 — Survey distributed to California public hospitals (See attached document). [file 1756-0500-6-233-S1.pdf]

1. Introduction

Survey of California Public Hospital Endoscopy Centers

Dear Colleague:

We at the Center for Specialty Access & Quality based at San Francisco General Hospital and UCSF are carrying out a project to better understand the delivery of GI endoscopic procedures within California's healthcare safety net. As part of this project we are conducting this survey of GI endoscopic services provided to patients who utilize public hospitals in California. Our project is supported by a grant from the California Health Care Foundation.

Our objective is to learn about the GI endoscopic resources available to patients within the California healthcare safety net. All information collected from this survey will remain anonymous and no hospitals will be identified in any resulting publications. We would greatly appreciate it if you could complete the survey by May 22, 2010. Thank you very much in advance for your time and commitment to helping us on this very important project. We will be happy to share with you the results of this survey if you would drop us an email indicating this.

For this survey an endoscopy center is defined as a place where endoscopic procedures are performed either for diagnostic or therapeutic purposes for the patients that are provided medical care by your public/county hospital.

Sincerely,

Hal F. Yee, Jr, MD, PhD

Director, SFGH/UCSF Center for Specialty Access & Quality
Chief Medical Officer and Chief of Gastroenterology
San Francisco General Hospital and Trauma Center
Rice Memorial Distinguished Professor of Medicine
University of California, San Francisco

2. Endoscopy Center Efficiency Project

1. How are patients scheduled for an initial endoscopic procedure at your endoscopy center?

- Closed access (prior GI clinic appointment required for scheduling of endoscopy)
- Open access (direct referral from primary provider without a prior GI clinic appointment)

2. Do you utilize any of the following healthcare providers to perform endoscopic procedures at your endoscopy center? (Please check all that apply)

- Nurse practitioner (NP)
- Physician assistant (PA)
- Gastroenterology Fellow
- Surgeon
- Family Practice/Medicine/Surgery Resident

3. Did your endoscopy center perform and if so how many of the following GI procedures in 2009 (to your best approximation)?

	Procedure performed in 2009?	Number of procedures performed in 2009?
Colonoscopy		
Flexible sigmoidoscopy		
Upper endoscopy		
ERCP		
EUS		
Balloon assisted enteroscopy		
PEG		
Video capsule endoscopy		
Manometry/motility		
pH Monitoring/BRAVO		
Percutaneous liver biopsy		
Therapeutic paracentesis		

4. To your best approximation, what is the mean wait time between when a patient is scheduled for an endoscopic procedure and the procedure date (in days)? (If you do not measure this please respond N/A)

5. To your best approximation, what proportion (%) of your patients do not show up or cancel the day of their endoscopic procedure(s)? (If you do not measure this please respond N/A)

6. How many half days in 1 week does your endoscopy center perform procedures? (A half day is defined as 1 room performing procedures for 4 hours. For example, if your endoscopy center used 2 rooms for 4 hours each for 1 day a week this would be a total of 2 half days)

7. Please mark the performance measurements that you currently collect/record in your endoscopy center (Please check all that apply).

- % of procedures that start on-time
- No-show/cancellation
- Procedure duration
- Procedure volume
- Room turn-over time
- Wait time for procedure (i.e. time between when a procedure is scheduled and the procedure date)

8. Please mark the following quality indicators for colonoscopy that you currently record/measure in your endoscopy center (Please check all that apply).

- Adenoma detection (for screening colonoscopy)
- Bowel preparation
- Cecal intubation
- Complication
- Withdrawal time

9. How many total full time equivalents (FTEs) of the following are employed at your center for performing endoscopy? (If you do not employ the position listed please answer N/A)

Physician	
Registered nurse (RN)	
Licensed vocational nurse (LVN)	
Nurse Practitioner/Physician assistant	
Endoscope reprocessor	
Technician (i.e. individual who assists endoscopist with procedure)	
Clerical (i.e. schedulers of endoscopic procedures)	

10. Which of the following methods does your endoscopy center use to give a patient their pathology results after an endoscopic procedure?

- Letter from endoscopy center with results
- Telephone contact with results
- Return GI clinic appointment for the results
- Patient to follow-up with primary/referring MD
- Other

11. In the majority of your endoscopy cases who primarily administers sedation? (Please select only one)

- Anesthesiologist
- Gastroenterologist
- Nurse
- Nurse anesthetist

12. To your best approximation, what proportion (%) of your endoscopic procedures are performed with an anesthesiologist/nurse anesthetist?

13. To your best approximation, what are the 2009 costs for your endoscopy center for the following general categories of expenditures? (If you do not know please answer N/A)

Supplies (i.e. general, disposable items used)

Personnel

Equipment leases

Equipment purchases (i.e. endoscopes, endoscope reprocessor machines, non-disposable items)

14. Are your pre-procedure/preparation room (i.e. where a patient waits in a gown prior to their procedure) separate from your recovery room?

- Yes
- No

15. At any given time, what is the maximum number of patients that can occupy each of the following rooms?

Pre-procedure/preparation

Recovery
